# Notes and News

**Published:** 1903-09-26

**Authors:** 


### NOTES AND NEWS.

The Prince and Princess of Wales (grand president and lady grand president of the League of Mercy) have made the following appointments:—Princess Alice of Albany to be lady president of the Epsom-Esher district, Princess Victoria of Schleswig-Holstein to be lady president of the East Berks district, Princess Louise Augusta of Schleswig-Holstein to be lady vice-president of the South Kensington district, the Duchess of Somerset and Mr. Henry L. Jephson, L.C.C., to be lady president and president of the North Kensington district, Lord and Lady Decies to be president and lady president of the Isle of Thanet District, the Hon. Mrs. Leveson-Gower to be lady president of the Reigate district, Lieutenant-Colonel Sir Adelbert Cecil Talbot to be president of the Epsom-Esher district, Mr. J. A. Fyler, M.P., to be president of the Chertsey district, the Rev. Dr. Wood, headmaster of Harrow School, and Mrs. Wood to be president and lady president of the Harrow district, and Mrs. Carr-Gomm to be lady president of the Rotherhithe district.

October 10th will be Hospital Saturday in London this year.

The plague in Mauritius is of a very fatal character. Of the 39 cases quoted last 28 terminated fatally.

Dr. W. H. Allchin will deliver the Harveian Oration at the Royal College of Surgeons on October 19th at 4 p.m.

The cocaine habit is growing to such an extent amongst negroes that it seems likely to parallel the opium habit in the East.

Mr. Frederic Mocatta has laid the foundation-stone of a new hospital at Aix-les-Bains, to be erected to the memory of the late Dr. Brachet. He was well known to English visitors, who have largely subscribed to the memorial.

An association has been formed at Constantinople for the prevention of consumption.

Typhoid fever has broken out in Dundee to a somewhat alarming extent. The cause seems to be uncertain.

Sir William Broadbent will present the prizes and Mr. V. Warren Low will deliver the introductory address at the opening of the winter session at St. Mary's Hospital.

Mr. Haffkine has published a most interesting and instructive summarised report of the work done at the Bombay Plague Research Laboratory between 1895 and 1902 by himself and his assistants.

The use of the skiagraph is now becoming so popular that surgeons are not at all surprised at the arrival of the patient at the consulting-room with his skiagraph under his arm to assist in the diagnosis.

The Marquis of Zetland, President of the Mount Vernon Hospital for Consumption, Hampstead, will open the new east wing of the building on Tuesday, October 27th. This extension will provide additional accommodation for 45 patients, and has been specially designed for carrying out the hygienic treatment of consumption.

The next examination of the Sanitary Inspectors' Examination Board for certificates of qualification for appointment of sanitary inspector, or inspector of nuisances, under section 108 (2) (d) of the Public Health (London) Act, 1891, will be held in London on Tuesday, January 19th, 1904, and the four following days. Particulars will be forwarded on application to the Honorary Secretary, Wm. R. E. Coles, 1 Adelaide Buildings, London Bridge, London, E.C.

The annual exhibition of pathological specimens added to the museum of St. George's Hospital since last year will be on view from October 1st to October 17th. A description is attached to each specimen. This exhibition is an excellent idea, and one which should be followed by all hospitals, as material must be collected yearly at every hospital amongst which something is always likely to be found of interest to the intelligent post-graduate as well as to the unqualified student.

The man who "wants to work and is willing to work" has at last been found—in a workhouse. The Colchester Board of Guardians advertised for some one to take the post of boys' caretaker. Among the applicants for the post was a man who wrote: "I am single, active, and in good health, and would waive all claim to superannuation." His application seemed to the guardians to be worth consideration, but when they made further inquiries regarding the applicant, they found to their surprise that he was an inmate of the union. We do not know if he got the post, but in view of his existing position the generous offer to waive all claim to superannuation shows a fine spirit of humour.

